# A common evaluation framework for the African Health Initiative

**DOI:** 10.1186/1472-6963-13-S2-S10

**Published:** 2013-05-31

**Authors:** Jennifer Bryce, Jennifer Harris Requejo, Lawrence H Moulton, Malathi Ram, Robert E Black

**Affiliations:** 1 Department of International Health, The Johns Hopkins Bloomberg School of Public Health, Baltimore, 21205, USA

## Abstract

**Background:**

The African Health Initiative includes highly diverse partnerships in five countries (Ghana, Mozambique, Rwanda, Tanzania, and Zambia), each of which is working to improve population health by strengthening health systems and to evaluate the results. One aim of the Initiative is to generate cross-site learning that can inform implementation in the five partnerships during the project period and identify lessons that may be generalizable to other countries in the region. Collaborators in the Initiative developed a common evaluation framework as a basis for this cross-site learning.

**Methods:**

This paper describes the components of the framework; this includes the conceptual model, core metrics to be measured in all sites, and standard guidelines for reporting on the implementation of partnership activities and contextual factors that may affect implementation, or the results it produces. We also describe the systems that have been put in place for data management, data quality assessments, and cross-site analysis of results.

**Results and conclusions:**

The conceptual model for the Initiative highlights points in the causal chain between health system strengthening activities and health impact where evidence produced by the partnerships can contribute to learning. This model represents an important advance over its predecessors by including contextual factors and implementation strength as potential determinants, and explicitly including equity as a component of both outcomes and impact. Specific measurement challenges include the prospective documentation of program implementation and contextual factors. Methodological issues addressed in the development of the framework include the aggregation of data collected using different methods and the challenge of evaluating a complex set of interventions being improved over time based on continuous monitoring and intermediate results.

## Introduction

In 2007 the Doris Duke Charitable Foundation (DDCF) launched the African Health Initiative (AHI) “…to help catalyze a shift from the current public health focus on single-disease programs to an emphasis on strengthening health systems to effectively deliver integrated primary care to underserved populations.” Other papers in this supplement describe the AHI and the five Population Health Implementation and Training (PHIT) Partnerships [1-6]. Generating new knowledge of global significance is central to the achievement of the AHI goal and requires a systematic mechanism for comparing and contrasting experiences and results across the five different country settings. The aim of the Initiative is to inform implementation in each site throughout the project period and produce lessons that may be generalizable to additional countries. Since this cross-site learning objective was defined after the five partnerships had been approved for funding. DDCF created the Population Health Implementation and Training Data Collaborative to provide a forum for sharing ideas and generating new knowledge for the field. Collaborative members include staff from partnership teams, DDCF, and The Johns Hopkins Bloomberg School of Public Health in their role as “Data Coordinator” for the Data Collaborative. An external technical advisory group (TAG), composed of experts in health systems research, advises the Collaborative.

In this paper, we introduce the common evaluation framework developed by the Collaborative and used by each partnership to guide their measurement and reporting. The framework includes 1) a conceptual model depicting how partnership activities are expected to lead to improvements in population health; 2) sets of “core” and “common” metrics defining quantified measurements that will be produced by all or at least two partnerships; 3) guidelines for supporting documentation on the implementation of partnership activities, which are quantifiable, as well as other contextual factors that may affect implementation or intervention effectiveness in improving population health; 4) and procedures for developing and maintaining the Collaborative databases. We close with a summary of methodological challenges, lessons learned, and expectations for the future.

## A conceptual model for the AHI Data Collaborative

All public health programs are based on a set of assumptions that reflect an underlying conceptual model (sometimes called an impact model or model of change) that specifies the pathways through which program activities (inputs and processes) will lead to changes in intermediate variables (outputs and outcomes) and eventual impact on population health. The model guides the selection of metrics and supporting documentation and provides a road map for the analysis of progress and results. Each of the PHIT Partnerships is based on a unique conceptual model reflecting how they expect that their strategy for health system strengthening will result in public health impact in their setting. The PHIT Data Collaborative was established after these plans had been developed and funded, so a first step was to work together to develop a posthoc conceptual model consistent with existing frameworks.

Consistency with existing frameworks is important because it will allow the Collaborative to contribute more easily to global learning. The PHIT Data Collaborative conceptual model was reviewed by all TAG members to strengthen its relevance and generalizability.

**Figure 1:**
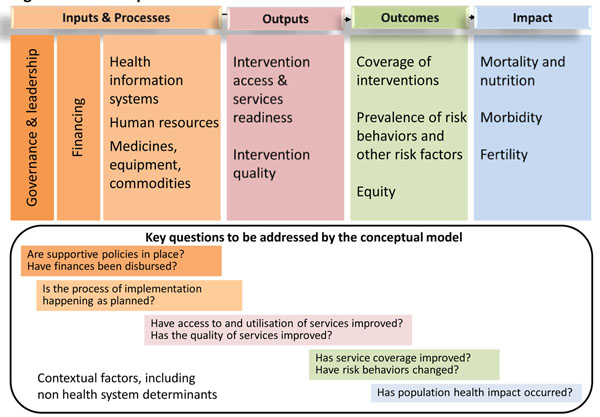
**A conceptual model for the AHI Collaborative.** Adapted from the CHeSS framework [12].

Figure 1 presents the current version of the conceptual model showing how the PHIT Partnership strategies are expected to lead to improvements in the health system and population health. The model is consistent with a suite of other evaluation frameworks developed to monitor progress toward Millennium Development Goals for women’s and children’s health [6] and to guide global efforts to scale up effective interventions [7], as well as with the WHO framework of six health systems building blocks (leadership and governance, health information systems, health system finance, health workforce, service delivery, and medical products, vaccines and technologies) [8,9]. This model is intended to be iterative and responsive to PHIT work and research findings throughout the implementation period. The evidence base is insufficient to support assumptions about specific health system strengthening activities, implementation intensities, or timelines needed to achieve health impact; it is precisely in this area that the AHI initiative hopes to make important contributions. This evaluation framework represents an important advance over its predecessors by including contextual factors and implementation strength as potential determinants of progress in the causal chain (Figure 1).

The first column in the model — inputs and processes — refers to a broad range of activities largely captured by five of the WHO six building blocks for health systems strengthening (with the exception of service delivery and associated service quality). Outputs refer to the short- and medium-term results of the inputs and processes and include health services utilization, readiness, and quality. Outcomes refer to increases in service coverage and improvements in health behavior that result from the earlier components in the model. Coverage is defined as the proportion of the population who require an intervention that actually receives it — an important component of behavior change. Impact refers to changes in health status, including mortality, nutrition, morbidity, and fertility.

Programs and interventions often fail to reach those who need them most, and overall progress in health outcomes or impact metrics can hide important disparities in progress by geographic location (e.g., urban/rural), gender, socioeconomic or ethnic group [10]. The Collaborative, therefore, incorporated equity into both the outcome and impact components of its conceptual framework. Core metrics in each of these components will be reported by wealth quintile as well as overall.

The questions at the bottom of the framework (Figure 1) reflect a stepwise approach to evaluating health systems reform and strengthening, and were adapted from the global “Country Health Systems Surveillance”(CHeSS) Initiative [11]. The questions are intentionally staggered from left to right, illustrating the need to achieve earlier steps before expected results can be obtained.

## Core and common metrics

Metrics related to the elements in the conceptual model were generated by reviewing the recommendations of the existing health systems frameworks described above and those developed for specific public health purposes (e.g., Millennium Development Goals target indicators [12], coverage metrics used by Countdown to 2015 for Maternal, Newborn and Child Survival [13] and disease-specific metrics recommended by Roll Back Malaria, Stop TB, UNAIDS, the Global Fund for AIDS, TB and Malaria, and others). Collaborative members consulted health systems and measurement experts — including members of the TAG — about their experiences in using these metrics and to generate additional alternatives. In addition, each partnership team mapped out all proposed activities by the six health system building blocks. The resulting matrix (Web annex 1) was used to guide the selection of metrics applicable to all the partnership strategies.

The Collaborative reviewed all proposed metrics and narrowed the list by applying the following criteria: 1) validity; 2) relevance to PHIT Partnership aims, public health importance, and sensitivity (likelihood of change as a result of partnership inputs and processes); 3) measurement feasibility; and 4) consistency with global standards. In addition, there was an effort to ensure that the set of metrics was amenable to linked hierarchical analysis and remained relatively small in number to reduce the reporting burden and to keep the Collaborative database manageable and focused.

Table 1 provides a summary of the Collaborative metrics. The only metric that was stipulated in the original Partnership agreements was the under-5 mortality rate, defined as the probability of dying before five years of age, expressed as a rate per 1,000 live births. All other metrics were agreed upon through the consultative process described here. Full definitions of core and common metrics are provided in Web Annex 2. Additional metrics may be defined over the course of the project. The Collaborative was not able to identify a core metric for the leadership and governance area that met the selection criteria. The construction of an index was discussed, but abandoned due to the challenges of defining comparable, quantitative variables across sites. (Table 1).

**Table 1:** Core and common metrics for the PHIT Data Collaborative by conceptual model component^1^

Inputs & Processes	Outputs	Outcomes	Impact
Governance and leadership: Financing: ■ **Total costs in intervention areas** Health Information systems: **■ Recent HMIS report available at facility** Human resources: ■ **Health workers per capita (physicians, nurses/midwives, pharmacy staff)** Medicines, Equipment, Commodities: ■ **Continuous stocks of essential commodities** **(Tracer equipment and commodities at health center level; Tracer medicines for all health facilities; Tracer medicines for health facilities providing specific services)**	Service access, readiness & quality: ■ Quality of child health care by providers ■ Service utilization	Coverage of services: ■ **Contraceptive prevalence rate** ■ **Antenatal care ( 1+ visits)** ■ **Intermittent preventive treatment for malaria in pregnancy (IPTp)** ■ **Skilled attendant at birth** ■ **C-section prevalence rate (urban, rural)** ■ **Exclusive breastfeeding** ■ **Childhood immunizations** ■ **Reported treatment of priority childhood illnesses** ■ **Vitamin A supplementation (2 doses)** ■ **Insecticide-treated net use** ■ TB treatment (DOTS) success rate ■ Antenatal care (4+ visits) ■ ART coverage ■ Post-natal care for mother ■ HIV testing for pregnant women ■ Stillbirth ratio: fresh/macerated ■ Unmet need for family planning Equity: ■ **Core coverage metrics reported by wealth quintile**	Mortality and undernutrition: ■ **Under 5 mortality rate** ■ **Cause of death distribution for under-fives in intervention areas** ■ Child undernutrition (height for age and weight for height) ■ Adult mortality rate ■ Neonatal mortality rate Morbidity: Fertility: ■ **Total Fertility Rate** Equity: ■ **Core impact metrics reported by wealth quintile**

^1^Core metrics shown in bold text

Methods for collecting and analyzing the core metrics would ideally be standardized across partnerships to produce results that are as comparable as possible. This aim could not be fully realized given that the research and evaluation plans of all partnerships had been established prior to the formation of the Collaborative. Adjustments have been made where feasible and affordable, and all partnerships are required to use methods that are replicable and meet current research standards. The core metrics for population health impact and outcomes all have population denominators, and will be measured at baseline and endline with additional intermediate measurements of coverage, nutritional status, and fertility where possible. Mortality will be measured using household surveys in Mozambique, Rwanda, and Zambia, and pre-existing demographic surveillance systems in Ghana and Tanzania. All coverage outcomes will be measured using household surveys with samples representative of the partnership and comparison areas. The definition and measurement of standard metrics becomes increasingly challenging as one moves toward the left of the conceptual model (Figure 1), and in these areas there are important differences in methods across the five sites which reflects the diversity of the partnership strategies. Examples of this methodological heterogeneity are provided in the cross-site papers included in this supplement [14,15]. Measurement of socioeconomic equity across the sites is based on standard approaches using principal component analyses of household assets to classify families into five equal groups, or wealth quintiles [16]. All core outcome and impact metrics will be disaggregated by wealth quintiles; individual partnerships may also elect to stratify results by age, gender and ethnic group.

## Supporting documentation

Analyses of quantitative metrics will have limited usefulness unless they are accompanied by clear descriptions of what was implemented by each partnership, how and at what levels of intensity and quality activities were implemented, and contextual factors that may have affected either how the strategy was implemented or its effectiveness. Information on these areas is provided by each partnership in their annual reports to DDCF. This continues to be a challenge because it is difficult to anticipate the types of information that will be relevant in advance.

Documentation of program implementation focuses on inputs and processes. It includes describing the intensity of program activities and how it varies across intervention areas. This information is needed for interpreting results obtained from the measurement of core and common metrics for outcomes and impact, highlighting the strengths and weaknesses of various partnership strategies. Details on program implementation processes are also important for determining how things are working, whether changes need to be made to specific program activities during the implementation period, and which activities are linked with improvements in coverage and population health.

Documentation of inputs will include the resources invested into the team strategies and encompass funding, strategies for procurement and distribution of commodities and project funds, partner coordination including relationship building with the Ministry of Health (MoH), harmonization, and the planning and policies supporting or hindering the implementation of the partnership strategies. Documentation of processes will include descriptions of the activities implemented by each partnership, (e.g., training and supervision activities, activities to build district-level management capacity, strengthening the supply chain management system, and improving information systems), and any other challenges or facilitating factors to successful project implementation.

Documentation of contextual factors (baseline and ongoing) is needed to identify positive and negative confounders that may affect the internal validity of the evaluation results, and potential effect modifiers that may enhance or diminish program implementation efforts and can affect the generalizability of the evaluation results [17]. Documentation of contextual factors is also essential for understanding why specific program activities may work in some settings and not others.

**Figure 2:**
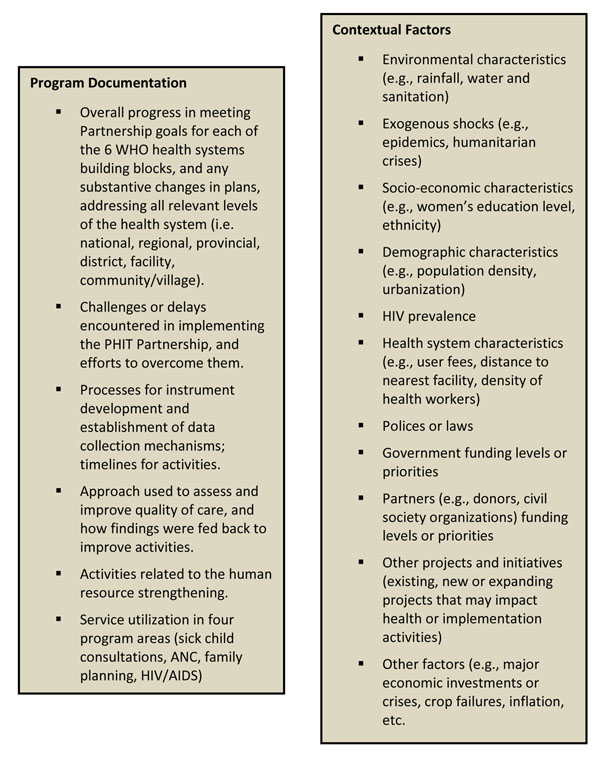
Topical list of supporting documentation being collected by the AHI Collaborative annually

Documentation of contextual factors involves reporting on key demographic, epidemiologic, socio-economic, political, and environmental factors likely to have a public health impact. The Collaborative has developed a standard set of quantitative measures for this purpose, supplemented through regular reporting on other contextual factors annually. Figure 2 summarizes the supporting documentation that partnerships update on an annual basis. The partnerships also provide narrative information annually on any relevant changes in policies or government leadership, funding levels and priorities of government and development partners, major economic changes or crises, and existing or new projects or initiatives that might impact their intervention and/or comparison areas (Figure 2).

## Data management and analysis

Each partnership had already developed its own systems for data acquisition, quality control, and subsequent data management prior to the development of the Collaborative. For efficiency purposes, the Collaborative agreed that each partnership will operate independently in all aspects of data collection and management and will work with the Data Coordinator to prepare and transmit the core and common metrics to the PHIT Data Collaborative repository.

Partnerships transmit two kinds of data to the Data Coordinator for the core and common metrics: summary data and the final data files from which these are derived, including the code that acts on the data files to produce the metrics. This information enables the Data Coordinator to verify the description and calculation of the metrics, examine the data for outliers, and produce queries regarding the submitted data. Of course, not all of these inputs are available for all metrics across the PHIT Partnerships — when metrics have been captured by externally organized surveys; such as a demographic and health survey (DHS) or multi-indicator cluster survey (MICS), only summary data are transmitted. What is required from the partnerships for each core and common metric, is an estimated standard error or confidence interval.

None of the data transmitted to the Data Coordinator will contain identifiable information; this specification has facilitated the data transmission agreements. Some countries have extensive regulations relating to external transmission of data recorded on their citizens. Issues related to data export have been minimized by using non-identifiable data and the establishment of careful procedures for continued control of the data release process by each partnership.

The Data Coordinator has established a password-protected SharePoint website for colocation of the metrics and documentation accumulated from each partnership. The data repository site is structured with a common area and partnership-specific silos. The common area includes information authorized by each partnership for viewing by the entire collaborative and general information about Collaborative activities. Data from each team are stored initially in partnership-specific silos and access is limited to partnership personnel. Once the partnership gives permission, the Data Coordinator moves the data from the partnership-specific silo to the common area. The Data Coordinator receives the documentation and data files from each partnership via email communication. After resolution of any queries resulting from quality assurance processes, the Data Coordinator uploads these files to either the common area or the partnership-specific silos, depending upon the level of access afforded to these files.

The primary data that will be used for cross-site analyses will be the core metrics (and standard errors) collected by each partnership at baseline and endline. Some metrics will be available at midline, and others (e.g., data from annual health facility surveys) may be captured on a more frequent basis. These will be organized in spreadsheets for easy viewing and will include both the overall and wealth quintile-specific metrics. Documentation data will also be available for identifying common themes, challenges, and successes. We anticipate there will be research efforts involving subsets of the partnerships that use not only the metrics, but analyze relationships among variables in the accumulated data, forming a pseudo-replication for strength of inference.

## Discussion: Challenges and lessons learned

The collaborative has learned valuable lessons over the past three years in its efforts to develop a common evaluation framework for the diverse set of five AHI PHIT Partnerships. We summarize the challenges here, and our efforts to address them in ways that will yield valid cross-site learning.

The first group of challenges relates to the fact that the evaluation framework was defined post-hoc, after the partnership plans for evaluation had been reviewed and funded through a highly competitive process. The first methodological challenge, therefore, was to agree on a conceptual framework that was acceptable to all groups as a starting point for adaptation to the specific strategies and activities planned in their country settings. This was particularly challenging given that creative innovation and diversity were explicitly encouraged during the proposal development process [1]. This “retrofitting” of the consensus AHI conceptual framework to the work of each partnership required repeated consultations over the course of three years. An early decision to adopt the WHO health systems building blocks framework resulted in a mapping exercise where all teams linked their activities to each of the building blocks (Web Annex 1). This enabled identification of areas of overlap of team activities, facilitated the selection of core and common metrics, and has led to cross-team conversations on specific issues (e.g., tool development for improving data quality, electronic patient records and other innovative technologies). We believe that these iterative efforts have contributed in important ways to clarifying the assumptions of the five partnerships and highlighting the different models being tested within the AHI portfolio.

There were challenges in defining a set of core metrics that met reasonable scientific criteria and yet reflected the central elements of all projects. We were largely successful in defining core metrics for outcomes (coverage and behaviors) and impact (health and nutrition), but found it difficult to do so for inputs, processes, and outputs because the five partnership strategies were heterogeneous and appropriately embedded in specific country contexts. For example, the economic components of the five evaluations varied widely, and the core metric in this area represents the least common denominator that was feasible for comparable measurement, i.e., the total per capita cost of health activities in the partnership intervention areas, broken out by the per capita incremental cost of partnership activities versus other costs. (Specific measures are the cost per capita of the PHIT program, cost per capita of total health system in intervention areas, and the PHIT contribution as percentage of total health system cost in intervention areas). The partnerships were also not able to come to consensus on a comparable indicator representative of the area of governance and leadership, and the collaborative therefore decided to collect information in this area through more qualitative documentation as part of the annual report process.

One ongoing challenge is to develop a workable system for data aggregation and analysis across sites. Procedures and permissions for data sharing across the multiple institutions involved in the Collaborative have been time-consuming because of varying regulations in the countries. Traditional multi-site evaluations often pool data collected in different sites for analysis; the Collaborative does not believe that this will be possible across the AHI partnerships because each partnership has planned distinct — although occasionally overlapping — interventions and approaches, and has proposed a specific research design that will not necessarily produce data directly comparable to those collected by other partnerships. We have also worked hard to develop strategies for maximizing the level of causal inference across sites given their different designs. For example, the Zambia team plans a randomized roll-out of its intervention through a step-wedge design [2] and Tanzania is implementing a cluster randomized controlled trial [3]. Randomization is the strongest approach for establishing a causal relationship between a study intervention and observed changes in population health. Plausibility designs as proposed by the other partnerships may be effective in capturing system-wide changes and their effects on population health.

These designs use comparisons of inputs, processes, outputs, outcomes, and impact in intervention areas with those in (non-randomized) non-intervention areas, and ecological dose-response analyses that take into account possible confounding, as well as mediating factors and effect modifiers, to assess project results. We expect that our efforts to collect and use supporting documentation will allow us to develop plausible inferences about the association between various types of health system strengthening activities and population impact. Opportunities for and the feasibility of specific types of cross-site analyses are reviewed at every annual meeting of the partnerships; the first cross-site research papers are included in this supplement [16,17].

Finally, we continue to be challenged by the need to design an approach for cross-site analysis of health system strengthening that will produce valid results despite the fact that the interventions are expected to evolve and change as a result of monitoring and intermediate project learning. The AHI partnerships are working in “real” country contexts, where successful activities may be adopted in other areas and separate programs with similar aims may be introduced in comparison areas. The Collaborative is working to address these issues by supplementing the tracking of core and common metrics with extensive documentation on program implementation and contextual factors in each of the five country sites for use in ensuring comparability and supporting interpretation of the results.

## Conclusions

The African Health Initiative aims to generate new knowledge and supporting evidence about how heath systems strengthening can lead to improvements in population health, drawing on experiences from five partnerships working in Ghana, Mozambique, Rwanda, Tanzania, and Zambia. The AHI PHIT Data Collaborative was formed after the partnerships had been designed and funded, and charged with developing a common evaluation framework that would contribute to the overall aim of AHI by fostering cross-site learning. This paper describes the results of these efforts, including an overall conceptual model outlining the pathways through which health systems strengthening can lead to increases in coverage for effective interventions and resulting health impact, a set of core metrics that are being measured by all partnerships, and a system for data compilation, quality checking, and analysis.

This process has highlighted the benefits of considering the requirements for evaluation and cross-site learning at the start of any multi-site initiative, and the importance of developing clear and fully specified conceptual frameworks as a prelude to field implementation. We have also learned that prospective evaluations require careful planning to ensure that activities and potential contextual factors are documented fully, and that designs must be sufficiently flexible to respond to changes over time in the programmatic landscape in both intervention and comparison areas.

The common evaluation framework for the AHI lays the foundation for generating important new knowledge about how health systems operate and how they can be strengthened to improve population health outcomes. The papers in this supplement provide a starting point for this program of learning, which will continue over the coming four years.

## List of abbreviations used

AHI: African Health Initiative; CHeSS : Country health systems surveillance; DDCF: Doris Duke Charitable Foundation; DHS: Demographic and health survey; MICS: Multiple indicator cluster survey; MoH: Ministry of Health; PHIT: Population Health Implementation and Training; TAG: Technical advisory group.

## Competing interests

The authors declare that they have no competing interests.

## Authors’ contributions

All authors have made substantial contributions to conception and design, have been involved in drafting the manuscript or revising it critically for important intellectual content, and have given final approval of the version to be published.

## Supplementary Material

Additional file 1 Definitions of Collaborative Core Metrics Click here for file

Additional file 2 Results of mapping Partnership activities onto the WHO six health systems building blocks. Click here for file

## Declarations

This article has been published as part of *BMC Health Services Research* Volume 13 Supplement 2, 2013: Improving primary health care to achieve population impact: the African Health Initiative. The full contents of the supplement are available online at http://www.biomedcentral.com/bmchealthservres/supplements/13/S2. Publication of this supplement was supported by the African Health Initiative of the Doris Duke Charitable Foundation.
