# Supplementary material for: A common evaluation framework for the African Health Initiative
Source: BMC Health Serv Res. 2013 May 31;13(Suppl 2):S10. doi: 10.1186/1472-6963-13-S2-S10 (PMC3668298; doi:10.1186/1472-6963-13-S2-S10)
Supplement: Additional file 1 — Definitions of Collaborative Core Metrics [file 1472-6963-13-S2-S10-S1.docx]

**Webannex 2. Definitions for Collaborative Metrics (Core and Common) and additional metrics considered**

**Table 2-1. Definitions of Collaborative Core Metrics**

| **Indicator Name and Type** | | **Indicator Definition** | **Numerator** | **Denominator** | **Data Source** | **Notes** |
| --- | --- | --- | --- | --- | --- | --- |
| **Public Health Impact** | | | | | | |
| 1 | Under-five mortality | Probability of dying before 5 years of age (expressed as a rate per 1,000 live births) | Number of under five deaths during time X | Number of live births during time X | Vital registration, household surveys, direct and indirect methods | MDG 4 indicator, Countdown indicator |
| 2 | Cause of death distribution for children under five | Percentage of deaths in children under five attributed to a specific cause | Number of under five deaths due to cause X | Total number of under five deaths | Verbal/social autopsy, interviews | Plans still in development. |
|  | *Child undernutrition* |  |  |  |  |  |
| 3 | Stunting prevalence | Percentage of children under five with chronic malnutrition (height for age z score less than -2 SD) | Number of children (0-4 years) that are stunted | Total number of children aged 0-4 | Standard anthropometric techniques; using WHO 2006 growth standards | Countdown indicator |
| 4 | Wasting prevalence | Percentage of children under five with severe acute malnutrition (height for weight z-score less than -2 SD) | Number of children (0-4 years) that are wasted | Total number of children aged 0-4 |  | Countdown indicator; |
| 5 | Total fertility rate | The average number of children that would be born to a woman if she were to live to the end of her childbearing years and bear children at each age in accordance with prevailing age specific fertility rates |  |  | Household surveys | UNICEF and UNFPA profiles |
| **Outcomes** | | | | | | |
| 6 | Antenatal care (at least one visit) | Percentage of women attended at least once during pregnancy by skilled health personnel for reasons related to the pregnancy | Number of women attended at least once during pregnancy by skilled health personnel (doctor, nurse, midwife or auxiliary midwife) for reasons related to the pregnancy in the X years prior to the survey | Total number of women who had a live birth occurring in the same period | Household surveys in representative samples of the population; incorporation of program data using standard methods for selected indicators | Countdown indicator, MDG 5 indicator |
| 7 | Intermittent preventive treatment for malaria in pregnancy | Percentage of women who received intermittent preventive treatment for malaria during their last pregnancy | Number of women at risk for malaria who received two or more doses of a sulfadoxine-pyrimethamine (Fansidar^TM^) to prevent malaria during their last pregnancy that led to a live birth | Total number of women surveyed who delivered a live newborn within the last two years |  | Countdown indicator, Roll Back Malaria indicator |
| 8 | Skilled attendant at birth | Percentage of live births attended by skilled health personnel | Number of live births to women ages 15–49 years in the X years prior to the survey attended during delivery by skilled health personnel (doctor, nurse, midwife or auxiliary midwife) | Total number of live births to women ages 15–49 years in the X years prior to the survey^c^ |  | Countdown indicator, MDG 5 indicator |
| 9 | Caesarean section rate (total, urban and rural where possible) | Percentage of live births delivered by Caesarean section | Number of live births to women ages 15–49 years in the X years prior to the survey delivered by caesarean section | Total number of live births to women ages 15–49 years in the X years prior to the survey |  | Countdown indicator |
| 10 | Contraceptive Prevalence rate | Percentage of women currently married or in union ages 15–49 that are using (or whose partner is using) a contraceptive method (either modern or traditional) | Number of women currently married or in union ages 15–49 years that are using (or whose partner is using ) a contraceptive method (either modern or traditional) | Total number of women ages 15–49 that are currently married or in union |  | Countdown indicator, MDG 5 indicator |
| 11 | Exclusive breastfeeding | Percentage of infants ages 0–5 months who are exclusively breastfed | Number of infants ages 0–5 months who are exclusively breastfed | Total number of infants ages 0–5 months surveyed |  | Countdown indicator |
|  | *Childhood immunizations* |  |  |  |  |  |
| 12 | Measles | Percentage of infants immunized with measles containing vaccine | Number of children ages 12–23 months who are immunized against measles | Total number of children ages 12–23 months surveyed |  | Countdown indicator/MDG 4 indicator |
| 13 | DPT3 | Percentage of infants who received three doses of diphtheria/pertussis/tetanus vaccine | Number of children ages 12–23 months receiving three doses of diphtheria/pertussis/tetanus vaccine | Total number of children ages 12–23 months surveyed |  | Countdown indicator |
|  | *Reported treatment of priority childhood illnesses* |  |  |  |  |  |
| 14 | Antimalarial treatment | Percentage of children ages 0–59 months with fever receiving any appropriate antimalarial drugs | Number of children ages 0–59 months reported to have fever in the two weeks prior to the survey who were treated with any appropriate antimalarial | Total number of children ages 0–59 months reported to have fever in the two weeks prior to the survey |  | Countdown indicator |
| 15 | Antibiotic treatment for pneumonia | Percentage of children ages 0–59 months with suspected pneumonia receiving antibiotics | Number of children ages 0–59 months with suspected pneumonia in the two weeks prior to the survey receiving antibiotics | Total number of children ages 0–59 months with suspected pneumonia in the two weeks prior to the survey |  | Countdown indicator |
| 16 | Oral rehydration and continued feeding | Percentage of children ages 0–59 months with diarrhoea receiving oral rehydration therapy and continued feeding | Number of children ages 0–59 months with diarrhoea in the two weeks prior to the survey receiving oral rehydration therapy (oral rehydration solution and/or recommended homemade fluids or increased fluids) and continued feeding | Total number of children ages 0–59 months with diarrhoea in the two weeks prior to the survey |  | Countdown indicator |
| 17 | Vitamin A supplementation (2 doses) | Percentage of children ages 6–59 months who received two doses of vitamin A during the calendar year | Number of children ages 6–59 months who received two doses of vitamin A during the calendar year | Total number of children ages 6–59 months |  | Countdown indicator |
| 18 | Insecticide-treated net coverage (U5) | Percentage of children ages 0–59 months sleeping under an insecticide-treated mosquito net | Number of children ages 0–59 months sleeping under an insecticide-treated mosquito net the night before the survey | Total number of children ages 0–59 months surveyed |  | Countdown indicator, Roll Back Malaria indicator |
| **Outputs** | | | | | | |
| 19 | Quality of child health care by providers | Proportion of children presenting to health facilities who are diagnosed with pneumonia, diarrhea, or malaria who are prescribed treatment correctly | The number of children presenting to health facilities who are diagnosed with pneumonia, diarrhea, or malaria who are prescribed treatment correctly | The total number of facilities | Health facility assessments; routine administrative records | Agreed this would be “common” rather than “core”; Data Coordinator has asked that this decision be reconsidered. |
| 20 | Service utilization | Monthly service volume by age in 1^st^ level facilities and from community based providers for selected programs in intervention and comparison areas. Services measured could include all contacts (rather than first-visit-for episode or first-for service), or be program specific. |  |  | Health facility assessments; routine administrative records | Teams are working with the Data Coordinator on developing a common definition and measurement approach across sites. |
| **Inputs and processes** | | | | | | |
| 21 | Total costs in intervention areas | Total costs in intervention areas plus incremental cost of implement PHIT Partnership strategy per capita in the intervention area |  |  |  | Standard approach for economic analysis |
| 22 | Recent HMIS report available at facility | Percentage of facilities which can produce for inspection the HMIS report that includes their data for the previous year | Number of facilities which can produce for inspection the HMIS report that includes their data for the previous year | Total number of facilities inspected | Health facility assessments |  |
| 23 | Health workers *per capita* (by cadre) | Ratio of health workers to population in the PHIT intervention area, by cadre and training status | Number of health workers at a given time in a given location | Total population in the same geographical location | Routine administrative records | WHO toolkit indicator; Data Coordinator will work with the teams on further defining this indicator. |
| 24 | Continuous stocks of essential commodities | Percentage of health facilities that have all tracer medicines and commodities in stock and prior to their expiration dates: on the day of the visit and in the last three months | The number of facilities with the selected tracer drugs in stock (present and non-expired) on the day of the visit and in the last three months | The total number of facilities | Health facility assessments; routine administrative records | WHO toolkit indicator; see tables B1a, B1b, and B1c for more details |

**Table 2-1a. Continuous stocks of essential commodities: Tracer medicines for all health facilities (health centers and above**)

| **Topic area** | | **Proposed item** |
| --- | --- | --- |
| ***Infectious diseases*** | | |
| 1 | Pneumonia | 1^st^ line antibiotic (child) |
| 2 | Malaria | ACTs (child and adult) |
| 3 | Diarrhea | Low osmolarity ORS + zinc (child) |
| 4 | Nutrition | Ready to use therapeutic fluids |
| ***Prevention/family planning*** | | |
| 5 | Vaccine-preventable | DPT3 (child) |
| 6 | Vaccine-preventable | TT vaccine (delivered to the mother for the benefit of the child) |
| 7 | Family planning | Oral or injectable contraceptive (adult) |
| 8 | Maternal health | 1^st^ line uterotonic (adult) |
| ***Chronic diseases*** | | |
| 9 | Hypertension | Anti-hypertensive (adult) |

**Table 2-1b. Continuous stocks of essential commodities: Tracer medicines for health facilities providing specific services**

| **Topic area** | | **Proposed item** |
| --- | --- | --- |
| *Health facilities providing TB services* | | |
| 1 | TB | 1^st^ line combination tablet (adults) |
| *Health facilities providing HIV services* | | |
| 2 | HIV | 1^st^ line treatment (adults) |

**Table 2-1c. Continuous stocks of essential commodities: Tracer equipment and commodities at health center level**

| **Topic area** | | **Proposed item** |
| --- | --- | --- |
| 1 | HIV | HIV test availability (all test kits needed for the national algorithm) |
| 2 | HIV/FP | Condoms |
| 3 | Hypertension | Sphygmomanometer (blood pressure cuff) and stethoscope |
| 4 | Pneumonia | Watch or timer or stethoscope |
| 5 | Vaccine –preventable | Refrigerator/cold box; Sterilization equipment |
| 6 | Nutrition – child | Infant/child weighing scale, MUAC |
| 7 | Nutrition – maternal | Tests for hematocrit or hemoglobin |
| 8 | Delivery care | Neonatal ambubag |

**Table 2-2. Definitions of Common Collaborative Metrics**

| **Indicator Name and Type** | | **Indicator Definition** | **Numerator** | **Denominator** | **Data Source** | **Will be measured and reported in:** |
| --- | --- | --- | --- | --- | --- | --- |
| **Public Health Impact** | | | | | | |
| 1 | Adult mortality rate | The probability of dying between ages 15 and 60 (expressed as a rate per 1,000 people aged 15 to 60) | Number of deaths in persons between the ages of 15 and 60 years at time X | Total number of persons between the ages of 15 and 60 years at time X | Vital registration, household surveys, direct and indirect methods | Rwanda, Zambia, Tanzania, Ghana |
| 2 | Neonatal mortality rate | The probability of dying in the first 28 days of life (expressed as a rate per 1,000 live births) | Number of deaths in the first 28 days of life at time X | Total number of live births at time X |  | Rwanda, Tanzania, Ghana |
| 3 | Cause of death distribution in adults | Percentage of deaths in adults under five attributed to a specific cause | Number of adult deaths due to cause X | Total number of adult deaths | Verbal/social autopsy, interviews | Zambia, Tanzania |
| **Outcomes** | | | | | | |
| 4 | Antenatal care (4+ visits) | Percent of women attended at least four times during pregnancy by any provider (skilled or unskilled) for reasons related to the pregnancy | Number of women attended at least four times during pregnancy by any provider (skilled or unskilled) for reasons related to the pregnancy in the X years prior to the survey | Total number of women who had a live birth occurring in the same period | Household surveys in representative samples of the population; incorporation of program data using standard methods for selected indicators |  |
| 5 | Postnatal care for the mother | Percentage of mothers who received a postnatal care visit within two days of childbirth | Number of women who received a postnatal care visit within two days of childbirth (regardless of place of delivery) | Total number of women ages 15-49 years with a last live birth in the x years prior to the survey (regardless of place of delivery) |  | Tanzania, Ghana, Zambia |
| 6 | TB treatment (DOTS) success rate | Proportion of new smear-positive TB cases registered under DOTS in a given year that successfully completed treatment whether with bacteriological evidence of success (“cured”) or without (“treatment completed”) | Number of new smear-positive TB cases registered under DOTS in a given year that successfully completed treatment whether with bacteriological evidence of success | Total number of new smear-positive TB cases registered under DOTS in a given year |  | Stop TB/MDG6/CHeSS platform indicator; Rwanda, Zambia, and Ghana |
| 7 | ART coverage | Proportion of the people on ART who need them | Number of people on ARTs at time X | Modeled by Spectrum |  | Mozambique, Zambia |
| 8 | HIV testing for pregnant women | The proportion of pregnant women who are tested for HIV during antenatal care contacts | Number of pregnant women tested for HIV during antenatal care contacts | Total number of pregnant women attending antenatal care | Health facility surveys; household surveys in representative samples of the population | UNGASS indicator, Zambia, Mozambique |
| 9 | Stillbirth ratio | The ratio of fresh to macerated stillbirths |  |  | Health facility surveys | Tanzania and Zambia |
| 10 | Unmet need for family planning | Percentage of women who are currently married or in union that have an unmet need for contraception | Number of women who are currently married or in union that are fecund and want to space their births or limit the number of children they have but that are not currently using contraception | Total number of women who are currently married or in union | Household surveys in representative samples of the population; incorporation of program data using standard methods for selected indicators | Countdown indicator, MDG 5 indicator. Mozambique, Ghana, Tanzania, Zambia |
| **Outputs** | | | | | | |
| **Inputs and processes** | | | | | | |
