# Supplementary material for: A common evaluation framework for the African Health Initiative
Source: BMC Health Serv Res. 2013 May 31;13(Suppl 2):S10. doi: 10.1186/1472-6963-13-S2-S10 (PMC3668298; doi:10.1186/1472-6963-13-S2-S10)
Supplement: Additional file 2 — Results of mapping Partnership activities onto the WHO six health systems building blocks. [file 1472-6963-13-S2-S10-S2.docx]

**Webannex 1. Results of mapping Partnership activities onto the WHO six health systems building blocks**

| **PHIT Partnership** | **Governance and Leadership** | **Financing** | **Health Information Systems** | **Human Resources** | **Medicines, Equipment, Commodities** | **Service Delivery** |
| --- | --- | --- | --- | --- | --- | --- |
| **Mozam-bique** | - Ensure district management teams are fully staffed - Regular training and ongoing mentoring for district and provincial management teams to fulfill and improve routine management duties - Support development and financing of annual district and provincial annual operational plans - Include community leaders in semi-annual facility performance review meetings and annual district planning and funds allocation exercise - Improve province🡪district and district🡪facility oversight and Supervision | - Support development and financing of annual district and provincial annual operational plans - Mentoring for provincial and district administration and finance staff to improve use and reporting of funds | - Ongoing assessments and feedback to strengthen HIS accuracy and consistency - Training, mentoring and supervision for HIS managers, facility staff to improve data quality - Develop tools to facilitate use of routine data for identifying bottlenecks and guide decision making (i.e. Supervision guides, facility checklists, OR models) | - Ongoing updates to routine HR information system - Develop simulation and optimization models to identify bottlenecks and guide efficient allocation of health personnel - Training and ongoing supervision for district and facility staff on management and leadership, other targeted themes - Ongoing mentoring for provincial HR managers | - Develop simulation and optimization models to identify bottlenecks and solutions for medicine and commodity logistics systems - Mentoring for provincial pharmacy managers to improve system functioning - Training and supervision to support district and facility pharmacy staff | - Improve availability and acceptability of health services by more efficient personnel allocation, reducing outages of medicines/commodities, more integrated and efficient facility workflow, and providing directed financial inputs into the system |
| **Rwanda** | - Support the MoH to implement the Rwanda strategic health plan for rural districts: - Foster good governance and effective management through training and on the ground management support at the district, hospital, health center and community level; - Engage district health leaders in identifying resource gaps (see financing HSS activities); - Increase use of information technology and data in the delivery of healthcare with a robust quality assurance and monitoring & evaluation system and provision of training; - Build Rwandan capacity for effective monitoring, evaluation, research, and analysis of health related data at the Rwandan institutions of the Ministry of Health (MOH), National Institute of Statistics (NIS), and National University of Rwanda School of Public Health (NUR SPH)] and local health management levels. | - With the MoH, determine from the district work plan gaps in funding and identify both internal or external sources to fill those gaps (see also governance and leadership): - Identify resource needs to assure adequate infrastructure exists at both health centers and Hospitals; - Identify critical financial gaps for human resources and provide support to meet as much as possible MoH health center and Hospital staffing norms (assuring adequately compensated and highly trained personnel); - Strengthen a cost recovery system for comprehensive supply chain & procurement system for drugs, diagnostics and other commodities; - Strengthen existing mutual health insurance system to assure health care is accessible to all. - Establish a long-term financing scheme for a well-trained compensated cadre of community health workers (CHWs). - Provide financial support for indigent and vulnerable patients focusing on nutrition issues in under 5’s, HIV patients, TB patients, and indigent patients (monitor to provide evidence of cost and efficacy of approach to argue for sustainability). | - With the MoH, establish a robust M&E system for Hospital, Health Center, and Community health systems, including making the system electronic: - Strengthen the health facility’s and district level management’s ability to report and use routinely collected health for improvement and planning; - Identify HIS gaps and work with health management to design and implement additional data collection systems to strengthen utilization of data for ongoing quality improvement and interim program evaluation, allowing for mid-course corrections - Establish a primary care registry/basic electronic medical record (EMR); - Link the HIS at health centers with the health mutual insurance system. - Strengthen data quality, collection and utilization of monthly reporting of key indicators from CHWs through existing national monthly reports, registers. - Use a CHW household chart in southern Kayonza. | - Establish well-trained and adequately compensated cohort of health care providers (doctors, nurses, lab techs, social workers, CHWs…etc): - Strengthen human resources at all supported facilities through training, clinical mentoring and supportive supervision; - Use IMAI, IMCI, IMPAC, HIV, TB, Chronic Care, and IMEESC protocols at health centers - Monitor health care providers’ knowledge and ability to use approved clinical protocols; - As feasible, meet MoH staffing norms for hospitals and health centers (see MoH norms for specific numbers of doctors, nurses…etc) - Establish a network of trained, supervised and compensated CHWs of approximately 75 per health center in southern Kayonza; - Strengthen district level health management capacity (also see Governance and leadership HSS activities) - Build local and national capacity for effective monitoring, evaluation and research (locally and at MOH and Rwanda University School of Public Health). | - Strengthening of supply chain management through a district pharmacy system: - Build a comprehensive supply chain & procurement system for drugs, diagnostics and other commodities; - Improve management of district pharmacy and assure adequate staffing. - Establish an electronic system for stock control at the district pharmacy - Assure adequate infrastructure including space, shelves, and a transportation mechanism for drug deliveries to health facilities. - Ensure compliance with MOH established Essential drugs List for   - Hospitals;   - health centers;   - IMCI community; - Electronic stock monitoring at the health center level with dedicated staff for pharmacy services; - Training for medicine dispensing and stock management at all health facilities. - Ensure minimum equipment stocking at supported health centers (BP, stethoscopes, sterilization, scales etc) - Ensure minimum supplies for CHWs (tape measures, charts, other supplies). | - To implement the MoH norms in terms of services offered in rural health districts and ensure effective quality services accessible to all: - Support full district Hospital services and infrastructure (see Rwanda MOH norms) ; - Establish/strengthen full health center services at designated health centers:   - Children IMCI   - Adult IMAI   - Chronic care   - ART and HIV care   - TB services   - Women’ Health (deliveries + IMPAC)   - Pre-natal care   - VCT   - Family planning   - Nutrition services for U5 (plumpy nut), HIV (BMI<18.5), and Socio Economic cases   - Nutrition of Hospitalized patients   - Emergency and wound care   - Simple Hospitalizations   - Social consult services - Establish Health Center infrastructure (water, electricity, maternity ward, consult room space, 10 hospital beds) - Implement CHW services (Community IMCI, Accompaniment of chronic disease and pregnancy). |
| **Tanzania-Ghana Health Partnership (Tanzania EMPOWER+ Project component)** | - Number of Council Assembly meetings with health agenda items - Number of district supervisors having undergone peer leadership training for supportive and effective supervision - Number of community demonstration and political engagement activities conducted - Number of community health committees convened - Number of meetings of governance committees (by level in the system) | - Alignment of Council health budgets with local burden of disease - Difference between budgetary plan and final utilization of funds - Per capita expenditure by component of the burden of disease and by health system level (district hospital/health centre/community) | - Proportion of facilities completing monthly reporting on time - Proportion of missing data in facility reports, by variable - Proportion of monthly reports with more than 5% discrepancy compared to patient records, by variable - Proportion of supervisors trained in data use - Proportion of communities with access to health facility data | - Number of Physicians, medical assistants deployed to district hospital/health centers - Number of community nurses deployed to community health service zone - Number of midwives deployed to health centers/community health service zones - Number of volunteers deployed to community health service zone - Number of health personnel receiving in-service training per cadre and training content | - Time in months since latest facility renovation (hospital/health centre/community health zone) - Availability of essential equipment (checklist including medical equipment, communication tools, transportation) - Availability of tracer drugs at quarterly intervals (= frequency of stock-outs) - Number of health workers trained in drug management | - Average patient waiting time at hospital/health centre/community health zone - Number of service days lost because of unavailable staff/facility closed |
| **Tanzania-Ghana Health Partnership (Ghana Essential Health Intervention Project component)** | - Number of District Assembly meetings with health agenda items - Number of district supervisors having undergone peer leadership training for supportive and effective supervision - Number of community demonstration and political engagement activities conducted (i.e., durbars) - Number of community health committees convened - Deployment of MPH graduates to district administrations | - Alignment of district health budgets with local burden of disease - Difference between budgetary plan and final utilization of funds - Per capita expenditure by component of the burden of disease and by health system level (district hospital/health centre/community) | - Proportion of facilities completing monthly reporting on time - Proportion of missing data in facility reports, by variable - Proportion of monthly reports with more than 5% discrepancy compared to patient records, by variable - Proportion of supervisors trained in data use - Proportion of communities with access to health facility data | - Number of Physicians, medical assistants deployed to district hospital/health centers - Number of community nurses deployed to community health service zone - Number of midwives deployed to health centers/community health service zones - Number of volunteers deployed to community health service zone - Number of health personnel receiving in-service training per cadre and training content | - Time in months since latest facility renovation (hospital/health centre/community health zone) - Availability of essential equipment (checklist including medical equipment, communication tools, transportation) - Availability of tracer drugs at quarterly intervals (= frequency of stock-outs) - Number of health workers trained in drug management | - Average patient waiting time at hospital/health centre/community health zone - Number of service days lost because of unavailable staff/facility closed |
| **Zambia PHIT** | - Hire, train, and mentor six district-based clinical quality improvement teams - Provide intensive mentoring and performance monitoring of facilities - Mentor existing district management staff through annual planning for district action plans; integrate PHIT activities in district action plans - Engage neighborhood health committees to conduct quarterly reviews with clinic managers of community-based health events and clinic performance | - Direct clinic inputs (equipment, supplies, renovations, clinic extender staff, community health workers) - Performance-based financing to districts in years 6 and 7 initially planned, currently removed due to budget cuts | - Electronic patient-level data produces routine HIS reports as well as clinic and community health worker performance reports - Clinic teams have quarterly quality improvement plans based on clinic performance reports | - 110 Clinic Extenders and 240 Community Health Workers - 18 district-based clinical quality improvement staff - Nurse Leadership training for quality improvement and district management staff at UAB - Ongoing clinical mentoring for district and facility based clinical staff - Management mentoring by health systems experts | - Back-up supplies of essential drugs and test kits - Mentoring for district pharmacy managers - District-based project pharmacy technician to support district pharmacy manager - Availability and correct use of drugs part of clinic performance reports | - Establish clear clinical protocols for primary health care, provide training and mentoring in these protocols, measure the care that is provided by electronic data entry, provide ongoing feedback on performance according to protocol, and mentor clinicians to improve the quality of care provided. |
